# Quantitative analysis of the MRI features in the differentiation of benign, borderline, and malignant epithelial ovarian tumors

**DOI:** 10.1186/s13048-021-00920-y

**Published:** 2022-01-22

**Authors:** Fuxia Xiao, Lin Zhang, Sihua Yang, Kun Peng, Ting Hua, Guangyu Tang

**Affiliations:** Department of Radiology, Shanghai Tenth People’s Hospital of Tongji University, 301 Middle Yanchang Road, Shanghai, 200072 China

**Keywords:** Ovarian neoplasms, Magnetic resonance imaging, Differential diagnosis

## Abstract

**Objective:**

This study aims to investigate the value of the quantitative indicators of MRI in the differential diagnoses of benign, borderline, and malignant epithelial ovarian tumors (EOTs).

**Materials and methods:**

The study population comprised 477 women with 513 masses who underwent MRI and operation, including benign EOTs (BeEOTs), borderline EOTs (BEOTs), and malignant EOTs (MEOTs). The clinical information and MRI findings of the three groups were compared. Then, multivariate logistic regression analysis was performed to find the independent diagnostic factors. The receiver operating characteristic (ROC) curves were also used to evaluate the diagnostic performance of the quantitative indicators of MRI and clinical information in differentiating BeEOTs from BEOTs or differentiating BEOTs from MEOTs.

**Results:**

The MEOTs likely involved postmenopausal women and showed higher CA-125, HE4 levels, ROMA indices, peritoneal carcinomatosis and bilateral involvement than BeEOTs and BEOTs. Compared with BEOTs, BeEOTs and MEOTs appeared to be more frequently oligocystic (*P* < 0.001). BeEOTs were more likely to show mild enhancement (*P* < 0.001) and less ascites (*P* = 0.003) than BEOTs and MEOTs. In the quantitative indicators of MRI, BeEOTs usually showed thin-walled cysts and no solid component. BEOTs displayed irregular thickened wall and less solid portion. MEOTs were more frequently characterized as solid or predominantly solid mass (*P* < 0.001) than BeEOTs and BEOTs. The multivariate logistic regression analysis showed that volume of the solid portion (*P* = 0.006), maximum diameter of the solid portion (*P* = 0.038), enhancement degrees (*P* < 0.001), and peritoneal carcinomatosis (*P* = 0.011) were significant indicators for the differential diagnosis of the three groups. The area under the curves (AUCs) of above indicators and combination of four image features except peritoneal carcinomatosis for the differential diagnosis of BeEOTs and BEOTs, BEOTs and MEOTs ranged from 0.74 to 0.85, 0.58 to 0.79, respectively.

**Conclusion:**

In this study, the characteristics of MRI can provide objective quantitative indicators for the accurate imaging diagnosis of three categories of EOTs and are helpful for clinical decision-making. Among these MRI characteristics, the volume, diameter, and enhancement degrees of the solid portion showed good diagnostic performance.

## Introduction

Epithelial ovarian tumor (EOT) is the most common type in the classification of ovarian tumors and are categorized as benign (BeEOTs), borderline (BEOTs), and malignant (MEOTs) on the basis of histological results. Ovarian tumors remain the first indication for gynecologic surgery [1, 2]. Laparoscopic tumor exfoliation or unilateral ovariectomy can be performed if the mass is a small BeEOT [3–6]. Young patients with BEOTs can undergo conservative surgery to preserve fertility or maintain ovarian function [7–10], whereas patients with MEOTs require the radical resection of tumors, followed by adjuvant chemotherapy [10–14]. Thus, the accurate diagnosis of the preoperative subtype of EOTs is important for the patient’s therapeutic schedule and prognosis. This study aims to analyze the quantitative indicators of magnetic resonance (MR) image for the accurate diagnosis of EOTs and explore the weight of those features in the differential diagnoses of the three types of EOTs through multiple regression analysis.

## Material and methods

### Patients

All patients with EOTs who underwent preoperative MRI from our picture archiving and communication system (PACS) database and had pathological results between January 1, 2009 and August 31, 2018 were retrospectively recruited. The subjects consisted of 477 patients with 513 EOTs. A total of 441 women had one mass, and 36 women had two masses. The histological subtypes of EOTs are shown in Table 2. The population characteristics and biochemical examinations are shown in Table 3. The recruit tumors were categorized into the BeEOTs, BEOTs, and MEOTs groups on the basis of the pathological results. This retrospective study was approved by the institutional review board with the waiver of the informed consent.

### MRI technique

The MR images were acquired using the 3.0-T MR imaging unit (Magnetom Verio, Siemens Medical Solutions, Germany) by employing a pelvic phased-array coil. The following imaging sequences were performed: transverse nonfat-suppressed T2-weighted turbo spin-echo sequences (repetition time [TR], 4050 ms; echo time [TE], 84 ms; section thickness, 4 mm; field of view (FOV), 325 mm; matrix, 384 × 256; and number of excitations [NEX] 2), transverse nonfat-suppressed T1-weighted gradient-echo sequences (TR, 550 ms; TE, 13 ms; section thickness, 4 mm; FOV, 325 mm; matrix, 384 × 256; and NEX, 2), sagittal fat-suppressed T2-weighted turbo spin-echo sequences, and coronal nonfat-suppressed T2-weighted turbo spin-echo sequences. Then, dynamic contrast-enhanced MRI (DCE-MRI) with 3D fat-suppressed T1-weighted interpolated spoiled gradient-echo sequence with volumetric interpolated breath-hold examination was performed in the transverse, sagittal, and coronal planes at scanning delay times of 40 and 120 s after the bolus injection (2.5 mL/s) of gadopentetate dimeglumine (0.5 mol/L, Beijing Beilu Pharmaceutical Company) at a dose of 0.1 mmol/kg, followed by 50 mL saline flush through the antecubital vein.

### MR images analysis

Two radiologists who were blinded to the pathological results independently reviewed the MR images and collected the clinical information of the patients. The characteristics of MRI include volume of tumor, maximum diameter of tumor, septum thickness, volume of solid portion, volume ratio of solid portion, maximum diameter of solid portion, maximum diameter ratio of solid portion, number of cysts, peritoneal carcinomatosis, ascites, bilateral involvemen. The degree of tumor enhancement was divided into three categories as follows: mild enhancement (less than), moderate (equal than) and prominent enhancement (greater than), compared with that of uterine myometrium on the delayed enhancement images of MRI. The criteria of MRI were elaborated on the basis of several previously published terms (Table 1).

**Table 1:** Definition of MRI findings

Term	Reference	Definition	Measurement standard
Septum thickness	Timmerman et al. [15].	Thickness of septum or septa within a cystic tissue	If the septum is irregular, select the thickest focal area.
Volume of tumor	GAO Mei-chun [16]	–	The volume of tumors was estimated in PACS by measuring the area of the tumor on contiguous 3.0 mm thick transverse slices throughout the whole length of tumor by using manually drawn boundaries. The area was generated automatically, and the volume of tumors were calculated by multiplying the slice thickness with the sum of the tumor cross-sectional area (Cavalieri’s principle)
Volume of solid portion	Timmerman et al. [15].	As defined by the IOTA group, at MR imaging, solid tissue enhances after gadolinium chelate injection. Therefore, the solid tissue includes vegetation.	The method of measurement was the same as that of the “volume of tumor”.
Volume ratio of solid portion		The proportion of solid components in the total tumorous volume	=Volume of solid portion/Volume of tumor
Maximum diameter of tumor		The diameter of the largest level of the tumor	–
Maximum diameter of solid portion		The diameter of the largest level of the tumorous solid portion	–
Maximum diameter ratio of solid portion		The ratio of the maximum diameter of solid portion and the maximum diameter of tumor	=Maximum diameter of solid portion/Maximum diameter of tumor

### Statistical analysis

Statistical analysis was performed using the SPSS 20.0 (SPSS, Inc., Chicago, IL, USA). Continuous variables, such as patient’s age and serum carbohydrate antigen 125 (CA-125) level, were expressed as mean ± standard deviation. The kappa and intraclass correlation (ICCs) coefficients were calculated to assess the interobserver agreement between the two readers for imaging parameter analysis. A kappa value of 0.00–0.20, 0.21–0.40, 0.41–0.60, 0.61–0.80, and 0.81–1.00 indicated slight, fair, moderate, substantial, and almost perfect agreement, respectively [17]. An ICC value of 0.00–0.10 indicated virtually no agreement, and ICC values of 0.11–0.40, 0.41–0.60, 0.61–0.80, and 0.81–1.00 indicated slight, fair, moderate, and substantial agreement, respectively [18]. In order to identify significant differences in MR imaging parameters, population characteristics and biochemical examinations, the Kruskal-Wallis test was used for continuous variables and categorical data among three groups. Multivariate logistic regression analysis was performed using all qualitative and quantitative variables to find the independent diagnostic factors. The receiver operating characteristic (ROC) curves were used to evaluate the diagnostic performance of MR characteristics and clinical information in differentiating BeEOTs, BEOTs, and MEOTs. ROC analysis was performed using the Medcalc version 15.6 (MedCalc Software, Mariakerke, Belgium). A *P* value < 0.05 was considered statistically significant.

## Results

### Histological subtypes of EOTs

Table 2 shows the distribution of pathological subtypes of EOTs include serous tumours, mucinous tumors, endometrioid tumors, clear cell tumors, brenner tumours, serous-mucinous tumors. Among these histological subtypes, serous tumours and mucinous tumors accounted for the majority of all subtypes (total percentage was 84.49%). Meanwhile, there was a high proportion of serous tumours and mucinous tumors in each group (92.43, 77.55%,61.22%, respectively).

**Table 2:** The distribution of histological subtypes of EOTs

	Benign(*n* = 330)	Borderline(*n* = 49)	Malignant(*n* = 98)
Serous tumours	161(48.79)	27(55.10)	39(39.79)
Mucinous tumors	144(43.64)	11(22.45)	21(21.43)
Endometrioid tumors	2(0.61)	1(2.04)	19(19.39)
Clear cell tumors	0(0)	2(4.08)	18(18.37)
Brenner tumours	5(1.51)	1(2.04)	1(1.02)
Serous-mucinous tumors	18(5.45)	7(14.29)	0(0)

The number in parenthesis is the percentage

### Clinical evaluation

The population characteristics and biochemical examinations of the blood samples of 477 patients with 513 ovarian masses are demonstrated in Table 3. Their mean age was 52.36 ± 12.71 (range 18–86) years. A total of 208 (43.61%) women were premenopausal, and 269 (56.39%) were postmenopausal. Thirty-six (7.55%) patients had bilateral tumors, and 441 (92.45%) patients had unilateral tumors. The significant differences were obtained for all indicators, including age, postmenopause, CA-125 level, human epididymis protein 4 (HE4), and premenopausal and postmenopausal risk of ovarian malignancy algorithm (ROMA) indices.

**Table 3:** Population clinical characteristics and biochemical examinations of blood

	BeEOTs(n = 330, n^*^ = 347, n^※^ = 305)	BEOTs(n = 49, n^*^ = 50, n^※^ = 48)	MEOTs(n = 98, n^*^ = 116, n^※^ = 109)	*P* value
Age	48.20 ± 13.04	47.61 ± 17.14	56.44 ± 7.79	0.001
Postmenopausal				0.002
No	148(44.85)	26(53.06)	25(25.51)	
Yes	182(55.15)	23(46.94)	73(74.49)	
CA-125	19.92 ± 29.00	89.82 ± 191.24	523.92 ± 835.60	<0.001
HE 4	51.92 ± 16.91	100.12 ± 124.28	260.23 ± 239.23	<0.001
Premenopausal ROMA index	9.23 ± 6.14	9.36 ± 5.77	47.81 ± 35.52	<0.001
Postmenopausal ROMA index	11.08 ± 3.68	20.06 ± 15.98	57.95 ± 30.22	0.001

The case number of BeEOTs(n) is 330 (unilateral 313, bilateral 17) with 347 tumors(n^*^). The number of tumors with contrast enhanced MR imaging(n^※^) is 305

The case number of BEOTs(n) is 49 (unilateral 48, bilateral 1) with 50 tumors(n^*^). The number of tumors with contrast enhanced MR imaging (n^※^) is 48

The case number of MEOTs(n) is 98 (unilateral 80, bilateral 18) with 116 tumors(n^*^). The number of tumors with contrast enhanced MR imaging (n^※^) is 109

The number in parenthesis is the percentage

### Interobserver agreement

For all MR imaging variables, the interobserver agreement was good (ICC = 0.899–0.999, kappa = 0.932–0.978; Table 4).

**Table 4:** Interobserver agreement of MR imaging variables

MR Imaging Variables	Κ value	ICC(95%CI)
Volume of tumor	–	0.988(0.985–0.991)
Volume of solid portion	–	0.899(0.870–0.922)
Volume ratio of solid portion	–	0.982(0.976–0.986)
Maximum diameter of tumor	–	0.988(0.985–0.991)
Maximum diameter of solid portion	–	0.995(0.994–0.997)
Maximum diameter ratio of solid portion	–	0.999(0.998–0.999)
Enhancement degrees	0.965	–
Ascites	0.978	–
Peritoneal carcinomatosis	0.932	–

### MR image analysis

**Fig. 1:**
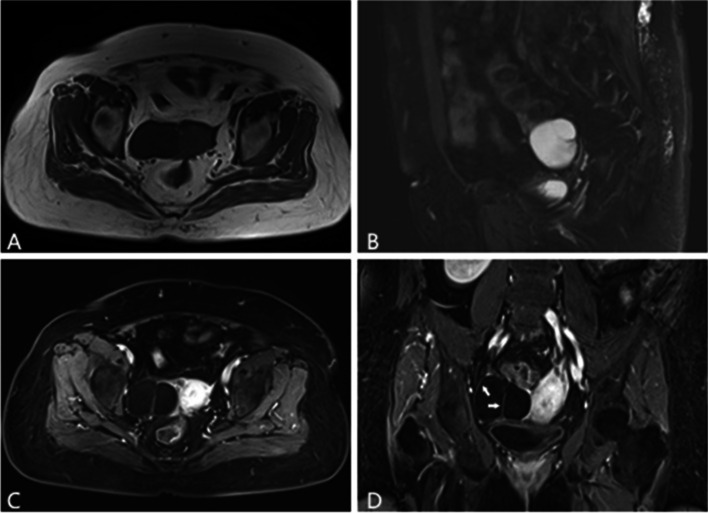
A 74-year-old woman with right serous cystadenoma. **A**–**B** Tumor with few loculi shows low and high signal intensities on T1WI and T2WI, respectively. The pelvis region has no peritoneal carcinomatosis and ascite. The thin wall and septum (arrows) in contrast-enhanced T1WI (**C**–**D**) exhibit mild enhancement

**Fig. 2:**
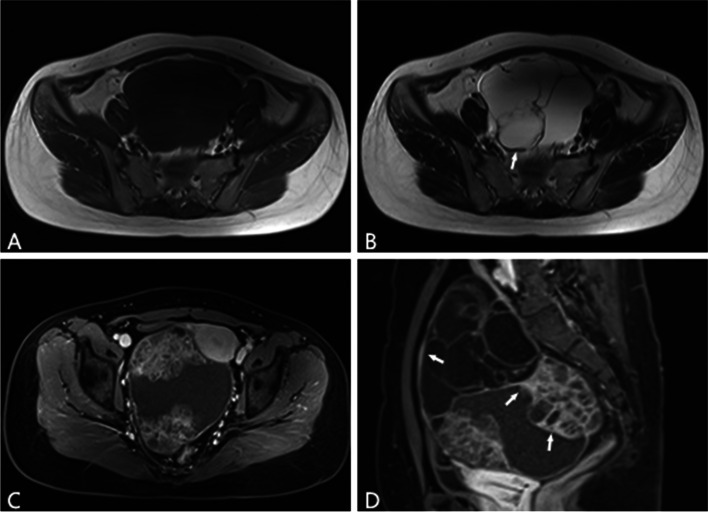
A 25-year-old woman with right mucinous borderline neoplasm. **A**–**B** Multilocular cystic mass with mild thickened capsule wall on the axial T1W and T2W images in the pelvis (arrow). **C**–**D** Prominent enhancement of the unevenly thickened capsule wall and septum on axial and sagittal contrast-enhanced T1W images with FS (arrows)

**Fig. 3:**
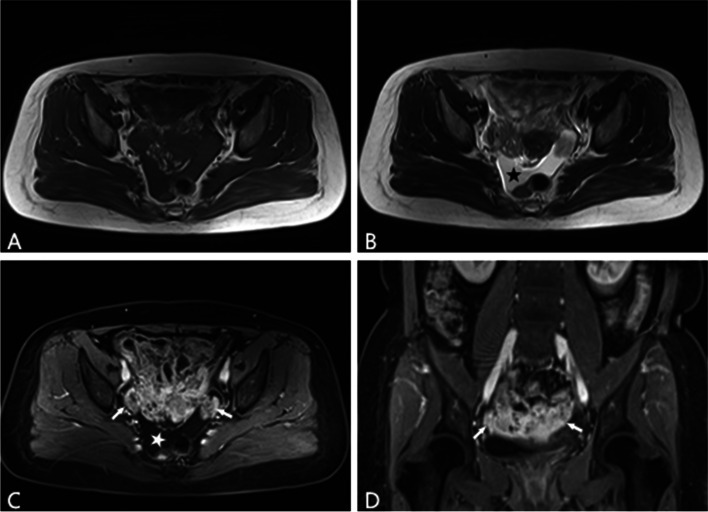
A 52-year-old woman with bilateral high grade of serous ovarian carcinoma. **A**–**B** Irregular solid mass on the bilateral ovarian regions with unclear boundaries present isointensity and slight hyperintensity signals on axial T1WI and T2WI, respectively. Ascite in rectum lacuna (pentastar) was found. **C**–**D** Axial and coronary contrast-enhanced fat-suppressed T1-weighted MR image shows markedly and unevenly enhanced solid component within complex solid and follicular mass in pelvis (arrows)

Table 5 shows the characteristics of the MR imaging findings in EOTs among benign, borderline, and malignant lesions by using univariate analysis. Compared with BEOTs, BeEOTs and MEOTs had less cysts (23/50, 46% vs 311/347, 89.63% and 88/116, 75.86%, *P*<0.001). Most BeEOTs had mild enhancement (290/305, 95.08% vs 16/48, 33.33% and 9/109, 8.26%, *P*<0.001) and less frequent ascites (75/330, 22.72% vs 27/49, 55.10% and 65/98, 66.33%, *P* = 0.003) than BEOTs and MEOTs. Peritoneal carcinomatosis was found in 24.49% (24/98) of MEOTs, 0% of BeEOTs, and 2.04% (1/49) of BEOTs (*P*<0.001), and bilateral involvement were more frequent in MEOTs (18.37%, 18/98) than in BeEOTs (6.06%, 20/330) and BEOTs (2.04%, 1/49) (*P* = 0.002, Figs. 1, 2 and 3). In quantitative MR imaging descriptors, BeEOTs usually showed thin-walled cysts and no solid component, but BEOTs often displayed irregular thickened walls and small amount of solid portion. MEOTs were more frequently characterized as completely solid or predominantly solid mass (*P*<0.001, Figs. 1, 2 and 3). No statistical difference was found among the three groups in terms of volume of tumor and maximum diameter of tumor (*P* = 0.058, *P* = 0.055, respectively).

**Table 5:** The difference of MRI parameters among three groups of EOTs

	BeEOTs(n = 330, n^*^ = 347, n^※^ = 305)	BEOTs(n = 49, n^*^ = 50, n^※^ = 48)	MEOTs(n = 98, n^*^ = 116, n^※^ = 109)	*P* value
Septum thickness	0.24 ± 0.11	0.53 ± 0.41	0.77 ± 0.34	<0.001
Volume of tumor	483.30 ± 883.11	1106.15 ± 2000.28	412.88 ± 674.36	0.058
Volume of solid portion	0.00 ± 0.00	57.23 ± 163.74	79.63 ± 120.08	<0.001
Volume ratio of solid portion	0.00 ± 0.00	9.58 ± 19.98	43.36 ± 37.49	<0.001
Maximum diameter of tumor	9.02 ± 6.19	12.44 ± 7.63	9.47 ± 4.50	0.055
Maximum diameter of solid portion	0.00 ± 0.00	2.52 ± 3.60	5.25 ± 3.03	<0.001
Maximum diameter ratio of solid portion	0.00 ± 0.00	22.84 ± 30.11	62.56 ± 33.19	<0.001
Bilateral involvement				0.002
No	310(93.94)	48(97.96)	80(81.63)	
Yes	20(6.06)	1(2.04)	18(18.37)	
Number of cysts				<0.001
<5	311(89.63)	23(46.00)	88(75.86)	
5–10	15(4.32)	3(6.00)	13(11.21)	
>10	21(6.05)	24(48.00)	15(12.93)	
Enhancement degrees				<0.001
Mild	290(95.08)	16(33.33)	9(8.26)	
Moderate	0(0)	4(8.33)	29(26.60)	
Prominent	15(4.92)	28(58.34)	71(65.14)	
Ascites				0.003
No	255(77.27)	22(44.90)	33(33.67)	
Yes	75(22.72)	27(55.10)	65(66.33)	
Peritoneal carcinomatosis				<0.001
No	330(100)	48(97.96)	74(75.51)	
Yes	0(0)	1(2.04)	24(24.49)

Multivariate logistic regression analysis was performed to obtain independent differential diagnostic factors. Results are shown in Table 6. The outcome revealed that volume of solid portion (*P* = 0.006), maximum diameter of solid portion (*P* = 0.038), enhancement degrees (*P* < 0.001), and peritoneal carcinomatosis (*P* = 0.011) were significant indicators for differentiate diagnosis of the three groups.

**Table 6:** Multivariate Logistic Regression of MR imaging parameters in EOTs

Covariate	Regression coefficient	Standard error	Wald	*P* value	*OR*
Age	0.001	0.022	0.002	0.964	1.00
Postmenopausal	0.851	0.597	2.029	0.154	2.34
Volume of tumor	0.000	0.000	0.154	0.695	1.00
Volume of solid portion	−0.008	0.003	7.520	0.006^*^	0.99
Volume ratio of solid portion	0.026	0.021	1.515	0.218	1.03
Maximum diameter of tumor	0.008	0.056	0.022	0.883	1.01
Maximum diameter of solid portion	0.453	0.218	4.328	0.038^*^	1.57
Maximum diameter ratio of solid portion	0.004	0.024	0.022	0.883	1.00
Bilateral involvement	1.076	0.881	1.492	0.222	2.93
Number of cysts	−0.236	0.244	0.930	0.335	0.79
Enhancement degrees	1.289	0.256	25.275	0.000^*^	3.63
Ascites	0.235	0.409	0.329	0.566	1.26
Peritoneal carcinomatosis	3.039	1.191	6.507	0.011^*^	20.88

^*^indicate a significant difference among three groups

Then, the diagnostic performance of MR imaging parameters, including volume of solid portion, maximum diameter of solid portion, enhancement degrees, peritoneal carcinomatosis, and their combination were assessed and compared using ROC analyses to differentiate two groups.

**Fig. 4:**
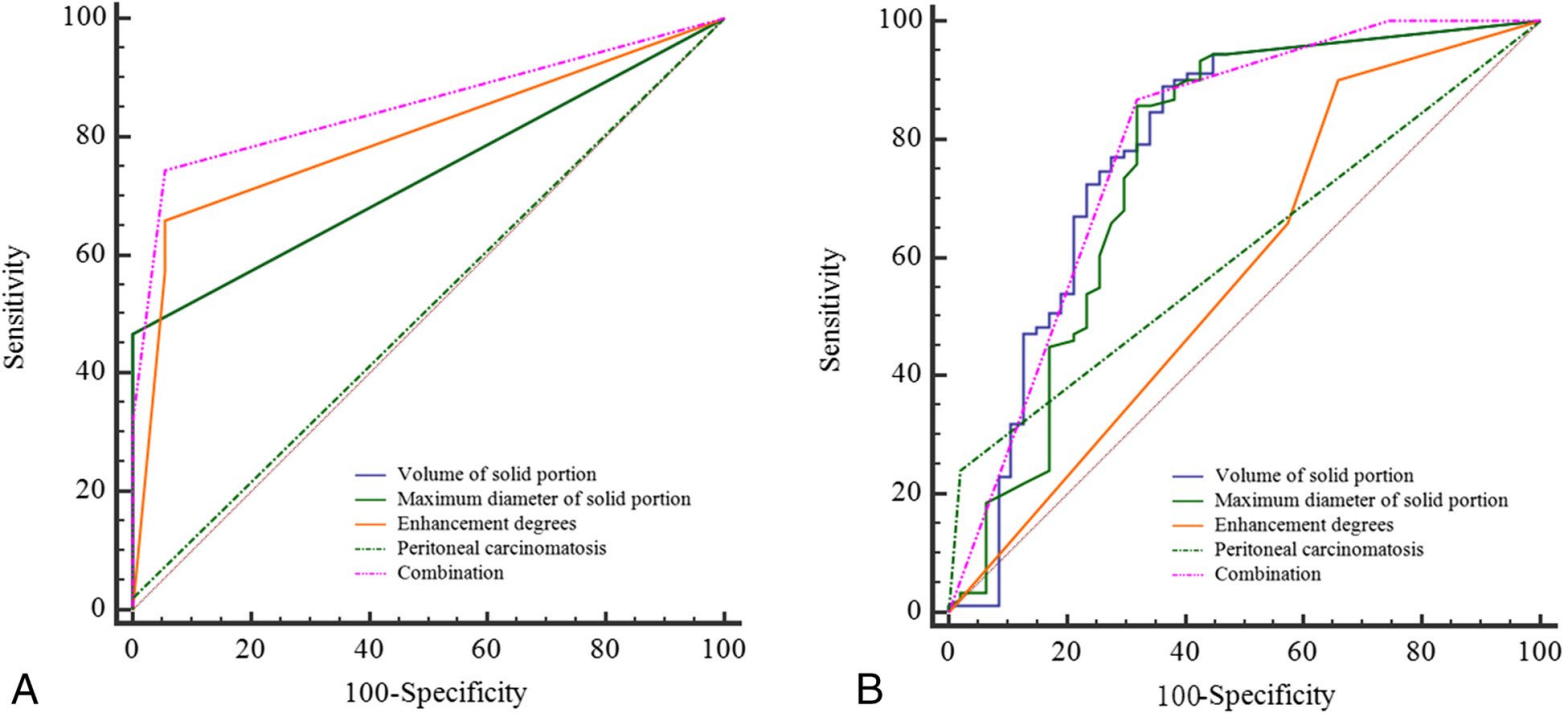
Receiver operating characteristic (ROC) curve analysis of MR imaging parameters, including volume of solid portion, maximum diameter of solid portion, enhancement degrees, peritoneal carcinomatosis, and their combination for discriminating BeEOTs and BEOTs (**A**) and BEOTs and MEOTs (**B**)

The area under the curve (AUC), sensitivity, specificity, positive predictive value (PPV) and negative predictive value (NPV) of this multivariate logistic regression model are shown in Table 7 and Fig. 4. In comparing BeEOTs and BEOTs, the image features of volume of solid portion, maximum diameter of solid portion, enhancement degrees, and the combination of four image features revealed moderate diagnostic value (0.74, 0.74, 0.8, 0.85, respectively), whereas peritoneal carcinomatosis showed low diagnostic value (0.51). Moreover, the above indicators except enhancement degrees (0.58) and peritoneal carcinomatosis (0.61) demonstrated moderate diagnostic value in BEOTs and MEOTs (0.78, 0.76,0.79, respectively).

**Table 7:** Receiver operating characteristic analysis of MR imaging parameters

		Cut-off value	Sensitivity	Specificity	AUC	PPV(%)	NPV(%)
Volume of solid portion	BeEOTs vs BEOTs	0.2	46.94	100.00	0.74	100.00	61.19
	BEOTs vs MEOTs	2.74	89.80	63.27	0.78	84.43	70.45
Maximum diameter of solid portion	BeEOTs vs BEOTs	0.4	46.94	100.00	0.74	100.00	61.19
	BEOTs vs MEOTs	2.2	86.73	67.35	0.76	85.47	67.35
Enhancement degrees	BeEOTs vs BEOTs	1	65.96	94.44	0.80	94.12	68.63
	BEOTs vs MEOTs	1	90.11	34.04	0.58	75.76	64.00
Peritoneal carcinomatosis	BeEOTs vs BEOTs	0	2.04	100.00	0.51	100.00	45.45
	BEOTs vs MEOTs	0	24.49	97.96	0.61	96.00	39.34
Combination	BeEOTs vs BEOTs	1	74.47	94.44	0.85	94.59	73.91
	BEOTs vs MEOTs	2	86.81	68.09	0.79	84.04	46.38

## Discussion

Adnexal masses, in general, are first found and evaluated using ultrasonography [19]. Nevertheless, in a prospective randomized trial in 2010, a consensus conference of the Society of Radiologists in Ultrasound proposed that establishing structured standards for adnexal cysts is needed [20]. To date, numerous scoring systems for preoperative mass discrimination have been developed [21]. Fernando Amor et al. [22, 23] proposed the Gynecologic Imaging Reporting and Data System (GI-RADS) to guide every imaging modality in describing and categorizing ovarian lesions in ultrasonography, but they did not specify the basis of classification and the imaging evidence for each category crucial to be recognized. This condition may be the reason that it is not recognized by radiologists to date. Therefore, authenticating their value on the basis of a large group of patients with EOTs is important.

In the past few decades, the MRI of the female pelvis has gained vast acceptance by gynecologists. In the literature, the accuracy of MR imaging in distinguishing malignant from benign complex adnexal masses ranges from 83 to 93% [24–28]. This result has been proven to be superior to CT in the assessment of complex and indeterminate ovarian tumors due to its excellent capacity for tissue characterization [29]. However, few studies have reported the structured standards for preoperative EOTs discrimination by using MR imaging.

The results of our study demonstrated some differences in the clinical data and the MRI findings of the three groups. Clinically, MEOTs often involved elderly patients and a high proportion of postmenopausal patients than the two other groups. In the biochemical index examination, CA-125 is the most common screening and monitoring marker of EOTs, but its sensitivity and PPV are not ideal because it can be increased in some benign non-neoplastic diseases [30]. HE4 was low in patients with benign ovarian diseases but highly expressed in patients with MEOTs [31–33]. Thus, ROMA is established and studied on the basis of the CA-125 and HE4 levels and postmenopausal status [25]. In our study, MEOTs showed higher CA-125 and HE4 levels and ROMA index than BeEOTs and BEOTs, which were consistent with the results of the above reports.

In the MR imaging findings, BeEOTs usually showed oligocystic, mild enhancement, and small probability of ascites. BEOTs often presented polycystic, prominent enhancement of parenchyma component, and high probability of ascites, similar to MEOTs. However, MEOTs showed bilateral involvement [34]. This phenomenon may indicate that the tumors grow on both sides or that the tumor on one side invaded the other ovary. By quantifying the weight of some MR imaging indicators, BeEOTs usually showed thin-walled cysts and no solid component. However, BEOTs often displayed irregular thickened walls and less solid portion, and MEOTs were frequently characterized as completely solid or predominantly solid mass [35]. Thus, the three groups of EOTs had some different objective characteristics on MR images.

Through multivariate logistic regression analysis, four imaging indicators, namely, volume of solid portion, maximum diameter of solid portion, enhancement degrees, and peritoneal carcinomatosis, were found significant in differentiating the three groups of EOTs. The enhancement of ovarian masses depends on the delivery and retention of contrast in the lesion. The vascular supply, capillary network, and leakage of contrast into the extravascular interstitial space contribute to the accumulation of contrast within the mass and great enhancement [36]. Our results showed that with the improvement of the subtype classification of ovarian tumors, increased solid components of tumors and prominent enhancement degrees were observed, which are in line with other reports [37]. The solid portion maybe had abundant tumor vascular supply [38], damaged basement membrane, and extracellular matrix. Consequently, MEOTs displayed prominent enhancement. MEOTs metastasize intra-abdominally with often numerous, superficial, small-sized lesions. This process is called peritoneal carcinomatosis. Previous literature has shown that peritoneal carcinomatosis may occur in BEOTs, but its incidence was evidently lower than that in MEOTs, which was consistent with our findings (2.0% vs. 24.49%). Serous carcinoma, particularly high-grade serous carcinoma, often appears as peritoneal carcinomatosis [39, 40]. The underlying mechanisms of interactions between MEOTs and peritoneal cells are incompletely understood. In addition, the mechanisms that enable tumor adhesion and growth probably involve cadherin restructuring on the epithelial ovarian cancer cells, integrin-mediated adhesion, and mesothelial evasion by mechanical forces driven by integrin–ligand interactions [41].

In terms of diagnostic performance, most quantitative indicators had a satisfactory performance and acceptable sensitivity and specificity, as shown by the multivariate analysis of MR imaging findings. The AUCs of these quantitative imaging indicators except peritoneal carcinomatosis in differentiating BeEOTs from BEOTs ranged from 0.74 to 0.853. However, the AUCs for differentiating BEOTs and MEOTs ranged from 0.579 to 0.791, indicating that the quantitative imaging measurement was useful for preoperative diagnosis and clinical decision-making. Therefore, this differentiation method can easily be generalized for use by all radiologists, regardless of their degree of expertise in pelvic imaging, as a means of improving report standardization.

Through the results of our study, MRI quantitative indicators provided relatively satisfactory results for the differentiation of EOTs. combined with MRI non-quantitative indicators and clinical indicators. Volume of solid portion, maximum diameter of solid portion, enhancement degrees, peritoneal carcinomatosis had high specificity in the identification of BeEOTs and BEOTs, which means that there was high probability of BeEOTs in multilocular cystic tumors with volume of solid portion less than 0.22 cm3, maximum diameter of solid portion less than 0.4 cm, no peritoneal metastasis and no or mild enhancement, which were consistent with the results of the previous report [42]. Volume of solid portion, maximum diameter of solid portion, enhancement degrees had high sensitivity in the identification of BEOTs and MEOTs, which means that there was high probability of MEOTs in multilocular cystic tumors with volume of solid portion more than 2.74 cm3, maximum diameter of solid portion mores than 2.2 cm and moderate or prominent enhancement. Unfortunately, some of above indicators had high sensitivity/specificity, but specificity/sensitivity was lower, When that happened, combination provided satisfactory sensitivity and specificity, which is integration of MRI features and clinical indicators. We need to combine all of the patient’s signs to provide greater accuracy in distinguishing of EOTs.

Several limitations were present in our study. First, some of the cases of pathological diagnosis were controversial. These cases included serous cystadenoma with focal borderline, which was categorized into BEOTs on the basis of the highest pathological grade. This practice narrowed the differences between the three groups or two groups to a certain extent. Thus, a detailed grouping and precise indicators on these tumors are necessary, which is crucial when deciding to opt for reasonable treatment [3–11]. Second, some cases were not performed using DW imaging and DCE-MRI in our early study. Thus, some other useful imaging features, such as ADC value and time–signal intensity curve, were not included for assessment. These factors will be considered in future research. Third, our results were based on the analysis of EOTs only and not available for other pathologic type masses, such as other types of neoplastic or non-neoplastic masses. Finally, all MR imaging examinations were performed in a single institution. The value of the indicators of these MRI features in the differentiation of three kinds of EOTs should be confirmed in a large prospective multicenter study.

In conclusion, this retrospective study has shown that the data of quantitative MR imaging indices can provide an objective basis for preoperative diagnosis and clinical decision-making. Among these indices, the volume of solid portion, maximum diameter of solid portion, enhancement degrees how good diagnostic performance. This result lay the foundation in proposing a standardized nomenclature for reporting the MRI findings of adnexal masses, which is especially useful for future artificial intelligence application in this field.

## Data Availability

All data generated or analysed during this study are included in this published article.

## Abbreviations

EOTs: Epithelial ovarian tumors
BeEOTs: Benign epithelial ovarian tumors
BEOTs: Borderline epithelial ovarian tumors
MEOTs: Malignant epithelial ovarian tumors
ROC: Receiver-operating characteristic
CA-125: Serum carbohydrate antigen 125
HE4: Human epididymis protein 4
ROMA: Risk of ovarian malignancy algorithm
MR: Magnetic resonance
AUC: Area under the curve
TR: Repetition time
TE: Echo time
FOV: Field of view
NEX: Number of excitations
DCE-MRI: Dynamic contrast-enhanced MRI
PACS: Picture Archiving and Communication System
IOTA: International Ovarian Tumor Analysis
ICCs: Intraclass correlation coefficients
NPV: Negative predictive value
PPV: Positive predictive value
GI-RADS: Gynecologic Imaging Reporting and Data System
DWI: Diffusion Weighted Imaging
ADC: Apparent Diffusion Coefficient
